# ApoA-IV improves insulin sensitivity and glucose uptake in mouse adipocytes via PI3K-Akt Signaling

**DOI:** 10.1038/srep41289

**Published:** 2017-01-24

**Authors:** Xiaoming Li, Fei Wang, Min Xu, Philip Howles, Patrick Tso

**Affiliations:** 1 National Local Joint Engineering Research Centre of Biodiagnostics and Biotherapy, The Second Affiliated Hospital of Medical College, Xi’an Jiaotong University, 157 W 5th Rd, Xincheng, Xi’an 710004, China; 2 Department of Pathology and Laboratory Medicine, Metabolic Diseases Institute, University of Cincinnati, 2180 E. Galbraith Road, Cincinnati 45237-0507, USA

## Abstract

Insulin resistance is a risk factor for type 2 diabetes mellitus. We investigated the effect of ApoA-IV on glucose uptake in the adipose and muscle tissues of mice and cultured 3T3-L1 adipocytes. We found that treatment with ApoA-IV lowered fasting blood glucose in both WT and diabetic *KKAy* mice by increasing glucose uptake in cardiac muscle, white adipose tissue, and brown adipose tissue through a mechanism that was partially insulin independent. Cell culture experiments showed that ApoA-IV improved glucose uptake in adipocytes in the absence of insulin by upregulating GLUT4 translocation by PI3K mediated activation of Akt signaling pathways. Considering our previous finding that ApoA-IV treatment enhanced pancreatic insulin secretion, these results suggests that ApoA-IV acts directly upon adipose tissue to improve glucose uptake and indirectly via insulin signaling. Our findings warrant future studies to identify the receptor for ApoA-IV and the downstream targets of PI3K-Akt signaling that regulate glucose uptake in adipocytes as potential therapeutic targets for treating insulin resistance.

Epidemiological studies have demonstrated that insulin resistance (IR) is associated with type 2 diabetes mellitus (T2DM), metabolic syndrome, and cardiovascular disease^1^,^2^. The loss of insulin sensitivity inhibits both the downregulation of hepatic gluconeogenesis and the stimulation of glucose uptake by myocytes and adipocytes, both which normally occur in response to an increase in the serum level of glucose^3^. In skeletal muscle, the translocation of the glucose transporter 4 protein (GLUT4) from intracellular compartments to the T-tubules and plasma membrane is required for glucose uptake^4^. The Rac1/GTPase and phosphatidylinositol 3 kinase (PI3K)-protein kinase B (Akt) signaling pathways stimulate GLUT4 translocation in skeletal muscle via separate mechanisms, both of which are dysregulated in IR^5^.

Apolipoprotein A-IV (ApoA-IV) is a major component of high-density lipoprotein and chylomicrons, both of which function in the transport of serum lipids^6^. ApoA-IV also plays an important role in generating the satiation signal via afferent vagal neurons following the consumption of dietary fat^7^. In our previous studies, we showed that treatment with exogenous ApoA-IV improved glucose homeostasis by suppressing hepatic gluconeogenesis and enhancing insulin secretion in both *KKAy* diabetic mice and *ApoA-IV*^−/−^ knockout (ApoA-IV-KO) mice with elevated serum glucose^8^,^9^. However, it is unclear whether ApoA-IV treatment enhances insulin sensitivity and glucose uptake in skeletal muscle, adipocytes, or other tissues.

We investigated the effects of ApoA-IV treatment on blood glucose levels and glucose homeostasis in adipocytes and muscle cells in mice. Our results showed that ApoA-IV reduced fasting blood glucose in *KKAy* obese diabetic mice and enhanced glucose uptake in the cardiac muscle and adipose tissue of wild-type (WT) mice. Cell culture experiments showed that ApoA-IV enhanced glucose uptake in mouse adipocytes via the PI3K-Akt mediated upregulation of GLUT4 translocation in the absence of insulin. According to our findings, the downstream effectors of ApoA-IV that mediate enhanced glucose uptake in adipocytes might represent potential therapeutic targets for the treatment of IR and T2DM.

## Materials and Methods

### Animals

All of our animal protocols were approved by the Institutional Animal Care and Use Committee of University of Cincinnati (OH, USA), and were performed according to National Institutes of Health Guide for the Care and Use of Laboratory Animals. Twelve-week-old male C57BL/6J mice, 8-week-old male *KKAy* mice (both from Jackson Laboratory, Bar Harbor, ME, USA), and 16-week-old male ApoA-IV-KO from a colony that we maintain at our institution^10^. A standard mouse diet (Teklad 7912, Harlan Laboratories, Indianapolis, IN, USA) was provided ad libitum. The mice were reared to 14–16 weeks of age before being used in our experiments, except where noted otherwise below.

### Insulin tolerance test

Recombinant mouse ApoA-IV (r-m-ApoA-IV) was expressed and purified as described previously^9^,^11^,^12^. After one hour of fasting, baseline blood samples were collected from the tail vein of fully conscious mice, after which an i.p. injection of 1 mg/kg r-m-ApoA-IV or saline was administered. After an additional 1 h of fasting, an i.p. injection of 0.5, 0.75, or 1 U/kg insulin (Humulin, Eli Lilly, Indianapolis, IN, USA) was administered, and blood samples were collected at 0.25, 0.5, 1, 2, 4, 6, and 9 h after insulin injection. The mice were denied access to food during the entire course of the experiment. Blood glucose concentrations were determined using a glucometer (Abbott Laboratories, Abbott Park, IL, USA), and blood insulin levels were measured using an ELISA (EMD Millipore, Billerica, MA, USA).

### Glucose uptake *in vivo*

After an overnight fasting period (22:00‒09:00), WT mice (*n* = 12) were pretreated with an i.p. injection of 1 mg/kg r-m-ApoA-IV, whilst additional WT mice were pretreated with an i.p. injection of saline (*n* = 24). Ninety minutes later, all of the mice were anesthetised by i.p. injection of 90 mg/kg sodium pentobarbital. The ApoA-IV pretreated mice were divided into two groups, consisting of the glucose-A-IV and basal-A-IV groups (*n* = 6 each group). The mice pretreated with saline were divided into four groups, consisting of the glucose-saline, glucose-Ins, basal-saline, and basal-Ins groups (*n* = 6 each group). Thirty minutes after inducing anaesthesia, a 50-μL blood sample was collected from the tail vein of each mouse for determining the baseline data (*t* = 0 min). Immediately after baseline blood collection, the mice received 0.33 mCi/kg [^3^H]2-deoxyglucose (Perkin Elmer, Waltham, MA, USA) as a glucose tracer via the retro-orbital sinus, which allowed the quantification of glucose uptake based on the level of intracellular tritium. The glucose-A-IV, glucose-saline, and glucose-Ins groups received tracer solution supplemented with 1 mg/kg glucose, and the basal-A-IV, basal-saline, and basal-Ins groups received tracer solution diluted in an equivalent volume of saline. The tracer solutions of the basal-Ins and glucose-Ins groups were also supplemented with 0.75 U/kg insulin. Additional 50-μL blood samples were collected from the tail vein at 5, 15, 25, 35, and 45 min after the glucose tracer was administered to determine serum glucose and insulin levels. The mice were denied access to food during the entire experiment, and data for mice from which a blood sample could not be collected at all of the time points were excluded from our analysis. After the final blood sample was collected, the anesthetised animals were sacrificed by cervical dislocation. The following tissues were collected from each mouse, and stored immediately in liquid nitrogen: EDL, SOL, TA, total muscle, BAT, WAT, CM, and WB. The GUR for each mouse was determined based on the level of [^3^H]2-deoxyglucose in tissue samples, which was assessed by dissolving the tissue in perchloric acid, followed by precipitation in Ba(OH)_2_ and ZnSO_4_, as described previously^13^. After adding 4 mL of scintillation fluid to each sample, [^3^H]2-deoxyglucose was quantified using a Beckman LS5000TD scintillation counter (Beckman Coutler, Fullerton, CA, USA).

In an additional glucose uptake experiment, two groups of WT mice and two groups of ApoA-IV-KO mice (*n* = 6 each group) were fasted overnight, and anesthetisation was performed as described above. Thirty minutes after inducing anaesthesia, the baseline blood sample was collected, and the glucose tracer with (glucose groups) or without (basal groups) 1 mg/kg glucose was administered to all of the mice via the retro-orbital sinus. No insulin or r-m-ApoA-IV was administered to either group. Additional blood samples were collected at 5, 15, 25, 35, and 45 min later. After final blood sample collection and euthanisation, the TA, EDL, SOL, CM, BAT, WAT, and WB samples were collected, and the serum glucose levels and tissue-specific GURs were determined as described above.

### Fibroblast cell culture and IMX induced differentiation to adipocytes

The 3T3-L1 murine fibroblasts (ATCC, Manassas, VA, USA) were seeded and propagated in DMEM supplemented with 10% FBS, as described previously^14^. Differentiation to adipocytes was induced by incubating the cells in DMEM supplemented with 10% FBS, 160 nM insulin, 0.25 μM dexamethasone, and 0.5 mM 3-isobutyl-1-methylxanthine (IMX) for 2 days. The cells were incubated for 2 additional days in the same medium lacking IMX. The cells were subsequently maintained in DMEM with 10% FBS and used as differentiated adipocytes between 8 and 10 day post-IMX induction.

### Glucose uptake in 3T3-L1 cells

The glucose uptake rates were measured in adipocytes using [^3^H]2-deoxyglucose as previously described^14^. The 3T3-L1 adipocytes (day 8 post-IMX induction) were washed twice with DMEM, after which the cells were incubated for 2 h at 37 °C in DMEM with no supplements. The cells were treated with 2.0, 5.0, 10, or 20 μg/mL r-m-ApoA-IV, wortmannin, or vehicle control for 0, 5, 15, 30, 60, and 120 min without insulin. The cells were washed twice with PBS and incubated for 5 min in 0.1 mM 2-deoxyglucose/[^3^H]2-deoxyglucose in PBS (1 μCi/mL). The cells were washed three times with ice-cold PBS, and solubilized using 0.4 mL of 1.0% SDS. After adding 4 mL of scintillation fluid, [^3^H]2-deoxyglucose was quantified using a Beckman LS5000TD scintillation counter. Nonspecific deoxyglucose uptake was measured in the presence of 20 μM cytochalasin B.

### ApoA-IV treatment for membrane protein isolation and analysis of Akt activation

The GLUT4 and Caveolin content in cell membrane fractions as well as dose-dependent effects of ApoA-IV on the phosphorylation and expression of Akt with and without wortmannin were determined by western blotting. The 3T3-L1 adipocytes (day 8 post-IMX induction) were starved in serum free DMEM medium overnight. To examine GLUT4 translocation, the cells were treated with 20 μg/mL r-m-ApoA-IV or vehicle control without insulin, and the cells were harvested by centrifugation at 0, 5, 15, 30, 60, and 120 min post-treatment. Membrane proteins were isolated from whole cell lysates using the Mem-PER Plus Membrane Protein Extraction Kit (Thermo Scientific, Waltham, MA, USA), according to the manufacturer’s protocol, and subjected to western blotting to detect GLUT4 and Caveolin. To examine the dose-dependent effects of ApoA-IV on the phosphorylation and expression of Akt, the cells were treated with 5, 10, 20, 50, and 100 μg/mL r-m-ApoA-IV or vehicle control for 30 min without insulin. The cells were harvested and incubated in TLB lysis buffer (20 mM Tris, 137 mM NaCl, 2 mM EDTA, 10% glycerol, 1% Triton X-100, 25 mM β glycerophosphoric acid disodium salt, ph7.4) to obtain whole cell lysates for western blotting to detect phospho-Akt, total Akt, and GAPDH (control). We also treated cells with 20 μg/mL r-m-ApoA-IV or vehicle control for 0, 5, 10, 20, 40, 60, 120, and 240 min to examine the effects of ApoA-IV on Akt expression and phosphorylation over time. The effect of wortmannin on ApoA-IV induced changes in Akt phosphorylation and expression were investigated by pretreating cells with 100 nM wortmannin or saline for 30 min before treatment with 20 μg/mL r-m-ApoA-IV for 40 min, and whole cell extracts were subjected to western blotting to detect phospho-Akt, total Akt, and GAPDH (control). We also treated cells with 100 nM wortmannin or saline for 30 min before treatment with 20 μg/mL r-m-ApoA-IV for 0, 0.5, 1, 2, 3, and 4 h to examine the effect of wortmannin on Akt expression and activation over time.

### Western blotting

The whole cell lysates from 3T3-L1 adipocytes were subjected to SDS-PAGE and western blotting using an anti-phospho-Akt antibody (Cell Signaling Technology, Danvers, MA, USA). The blots were stripped, and probed again using an anti-Akt antibody (Cell Signaling Technology). An anti-GAPDH antibody (EMD Millipore) was also used for an internal loading control for the whole cell lysates. Anti-GLUT4 and anti-caveolin (Cell Signaling Technology) antibodies were used to analyse aliquots of the membrane protein fractions (2.5 μg total protein). The Precision Plus Protein Standard (BioRad Laboratories, Hercules, CA, USA) was used for the molecular weight standard for all of the immunoblots. The results of at least three western blotting experiments were quantified using the TotalLab TL100 software (Sigma-Aldrich).

### Statistical analysis

Data are presented as the mean ± SEM of at least three independent experiments performed using triplicate samples (cell culture experiments) or four to six mice in each treatment group. Intergroup differences were compared using a one-way or two-way analysis of variance (ANOVA) followed by the Dunnett or Bonferroni test where appropriate, and two group difference was performed using t-test with the level of statistical significance set at *P* < 0.05.

## Results

### ApoA-IV improved insulin sensitivity in mice

**Figure 1:**
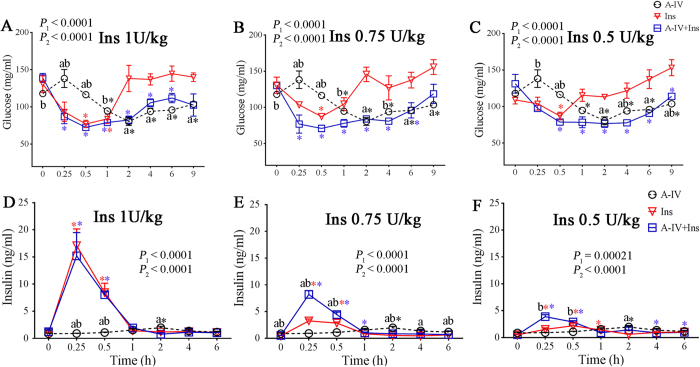
ApoA-IV improves insulin sensitivity in wild-type (WT) mice. Three groups of one-hour fasted WT mice (*n* = 4 to 6 per group) were pretreated with 1 mg/kg r-m-ApoA-IV (A-IV and A-IV + Ins groups) or saline (Ins group) for 1 h, followed by treatment with the dosage of insulin indicated in each panel (A-IV + Ins and Ins groups) or treatment with saline (A-IV group) for 1 h with continued fasting. Blood was collected from each mouse at 0, 0.25, 0.5, 1, 2, 4, 6, and 9 h after insulin treatment to determine (**A**–**C**) their blood glucose level between 0 and 9 h post-treatment and (**D**–**F**) their serum insulin level between 0 and 6 h post-treatment. The overall comparison of the datasets was performed using two-way ANOVA (*P*_*1*_ for A-IV vs. Ins; *P*_*2*_ for A-IV vs. A-IV + Ins), which was followed by the comparison of individual time points between two groups using the Bonferroni test (a indicates *P* < 0.05 for A-IV vs. Ins; b indicates *P* < 0.05 for A-IV vs. A-IV + Ins). Differences between the glucose and insulin levels at each time point relative to baseline were evaluated using one-way ANOVA followed by the Dunnett test (* indicates *P* < 0.05 for each group based on colour).

We examined the effects of recombinant mouse ApoA-IV (r-m-ApoA-IV) on glycaemic homeostasis in WT mice (C57BL/6J strain) using an insulin tolerance test (ITT). Treatment with various dosages of insulin following pretreatment with r-m-ApoA-IV at a dose of 1 milligram per kilogram body weight (1 mg/kg) significantly reduced the blood glucose level in WT mice beginning at 1, 0.5, and 2 h after treatment with 0.50, 0.75, and 1.0 U/kg insulin, respectively, compared to that observed in mice that received insulin only (*P* < 0.05; Fig. 1A‒C). In mice treated with insulin only, the level of blood glucose initially decreased for all the 3 doses of insulin injected. However, 1 h after insulin treatment, regardless of the dose of insulin administered, blood glucose rose to near baseline levels. By contrast, in the mice pretreated with r-m-ApoA-IV, blood glucose remained significantly lower than the baseline level (*P* < 0.05) for at least 6 h, regardless of the dosage of insulin administered, whereas the blood glucose level in the mice that received insulin only increased to near baseline levels (*P* ≥ 0.05) at 1 h (0.5 and 0.75 U/kg) or 2 h (1.0 U/kg) after insulin treatment. The insulin analysis showed that the serum level of insulin at 1‒6 h was near the baseline level for all of the treatment groups (Fig. 1D‒F), which suggests that the lower glucose levels observed between 0‒1 h post-treatment were the result of improved insulin sensitivity, whereas the glucose lowering effect between 1‒6 h was insulin independent (Fig. 1A‒C).

### ApoA-IV reduces blood glucose in *KKAy* diabetic mice

**Figure 2:**
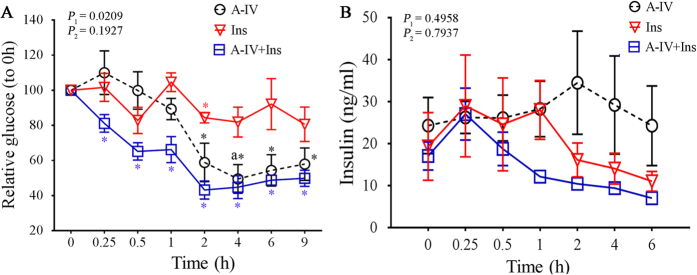
ApoA-IV improves insulin sensitivity in *KKAy* diabetic mice. Two-hour fasted *KKAy* mice (*n* = 4 per group) were treated with insulin (1 U/kg) following pretreatment with r-m-ApoA-IV (A-IV + Ins group) or saline (Ins group) for 1 h, or they received saline following the r-m-ApoA-IV pretreatment (A-IV group). Blood was collected from each mouse at 0, 0.25, 0.5, 1, 2, 4, 6, and 9 h after insulin treatment to determine (**A**) relative blood glucose (0‒9 h) and (**B**) serum insulin levels (0‒6 h). The overall comparison of the datasets was performed using two-way ANOVA (*P*_*1*_ for A-IV vs. Ins; *P*_*2*_ for A-IV vs. A-IV + Ins), which was followed by the comparison of individual time points between two groups using the Bonferroni test (a indicates *P* < 0.05 for A-IV vs. Ins; b indicates *P* < 0.05 for A-IV vs. A-IV + Ins). The glucose and insulin levels at each time point were compared to 0 h for each group using one-way ANOVA followed by the Dunnett test (* indicates *P* < 0.05 for each group based on colour).

We investigated whether ApoA-IV pretreatment would improve glycaemic response of *KKAy* obese diabetic mice in ITTs. In the *KKAy* mice pretreated with r-m-ApoA-IV, the relative blood glucose level was significantly lower (*P* < 0.05) than the baseline value from 0.25‒9 h after insulin treatment (Fig. 2A), whereas the mice that received saline following the r-m-ApoA-IV pretreatment had significantly lower relative blood glucose levels at 2‒9 h (*P* < 0.05, compared to baseline). By contrast, treatment with insulin alone did not significantly reduce the relative blood glucose level, compared to baseline, with the exception of the 4 h time point (*P* < 0.05). In addition, the *KKAy* mice that received both r-m-ApoA-IV and insulin had significantly lower relative blood glucose levels at 1, 2, 4, and 9 h, compared to those of mice that received insulin alone. The analysis of serum insulin levels showed that, although the level of insulin in mice that received saline following the r-m-ApoA-IV pretreatment was higher than that observed in the other groups, there was no statistically significant difference between the insulin levels in the three treatment groups at any of the time points (Fig. 2B). These data suggest that pretreatment with r-m-ApoA-IV increased insulin sensitivity in the *KKAy* mice. However, because a trend toward higher serum insulin was observed in the *KKAy* mice that received r-m-ApoA-IV alone, it was unclear whether the reduction in blood glucose was the result of an r-m-ApoA-IV-induced increase in the secretion of endogenous insulin in the *KKAy* mice or increased insulin sensitivity. The trend toward lower serum insulin in the A-IV + Ins group, compared with that of the Ins group, also suggests that insulin clearance might be affected by ApoA-IV.

### ApoA-IV increases glucose uptake in adipose tissue and cardiac muscle *in vivo*

**Figure 3:**
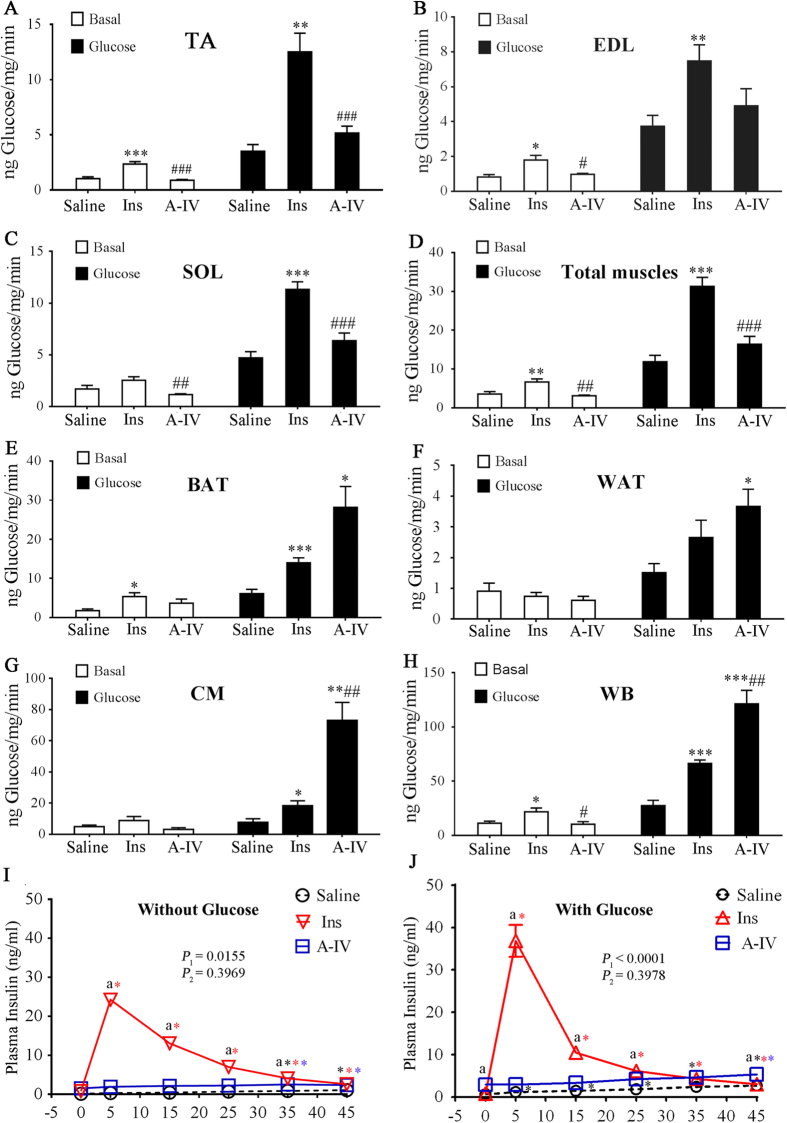
ApoA-IV increases glucose uptake in the tibialis anterior (TA), extensor digitorum longus (EDL), soleus (SOL), total muscle, intrascapular brown adipose tissue (BAT), visceral white adipose tissue (WAT), cardiac muscle (CM), and whole body (WB) of wild-type (WT) mice. After fasting overnight, WT mice (*n* = 12) were pretreated with 1 mg/kg r-m-ApoA-IV, whilst additional mice were pretreated with saline (*n* = 24), before inducing anaethesia for 30 min. The mice received 0.33 mCi/kg [^3^H]2-deoxyglucose in a solution containing 1 mg/kg glucose (glucose-A-IV and glucose-saline groups); 1 mg/kg glucose and 0.75 U/kg insulin (glucose-Ins group); or 0.75 U/kg insulin only (basal-Ins group), or they received [^3^H]2-deoxyglucose diluted in an equivalent volume of saline (basal-A-IV and basal-saline groups). The glucose uptake rates for the (**A**) TA, (**B**) EDL, (**C**) soleus, (**D**) total muscle, (**E**) BAT, (**F**) WAT, (**G**) CM, and (**H**) WB were determined (*P < 0.05, **P < 0.01, ***P < 0.001 for the glucose-A-IV and glucose-Ins groups vs. glucose-saline group or the basal-A-IV and basal-Ins groups vs. basal-saline groups; ^#^*P* < 0.05, ^##^*P* < 0.01, ^###^*P* < 0.001 for glucose-A-IV or basal-A-IV group vs. glucose-Ins or basal-Ins group, respectively). (**I** and **J**) Blood samples were collected at 0, 5, 15, 25, 35, and 45 min after [^3^H]2-deoxyglucose was administered to determine serum glucose and insulin levels (mean ± SEM). The overall comparison of the datasets was performed using two-way ANOVA (*P*_*1*_ for glucose-A-IV or basal-A-IV group vs. glucose-Ins or basal-Ins group, respectively; *P*_*2*_ for glucose-Ins or basal-Ins group vs. glucose-saline or basal-saline group, respectively), which was followed by the comparison of corresponding time points between two groups using the Bonferroni test (a indicates *P* < 0.05 for glucose-A-IV or basal-A-IV group vs. glucose-Ins or basal-Ins group, respectively; b indicates *P* < 0.05 for glucose-Ins or basal-Ins group vs. glucose-saline or basal-saline group, respectively). The insulin levels at each time point were compared to baseline for each group using one-way ANOVA followed by the Dunnett test (* indicates *P* < 0.05 for each group based on colour).

**Figure 4:**
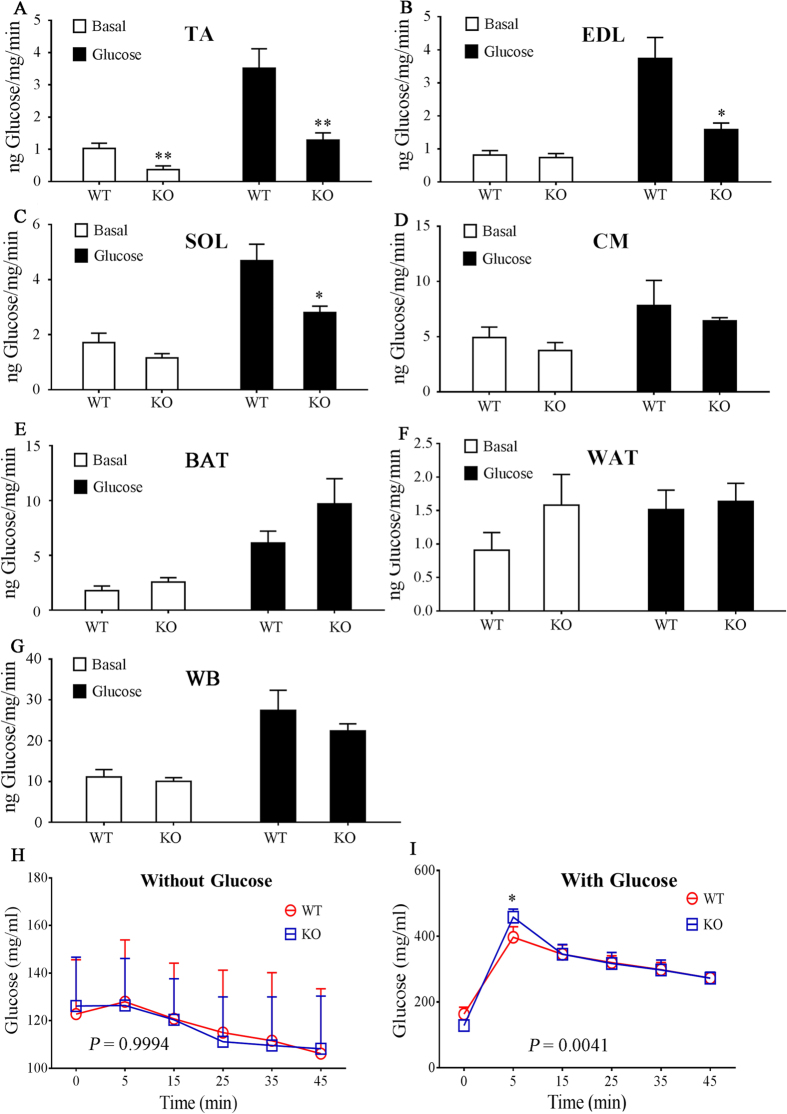
Glucose uptake in the tibialis anterior (TA), extensor digitorum longus (EDL), soleus (SOL), cardiac muscle (CM), intrascapular brown adipose tissue (BAT), visceral white adipose tissue (WAT), and whole body (WB) of ApoA-IV knockout (KO) mice. After fasting overnight, anaesthetised wild-type (WT) mice and KO mice were treated with [^3^H]2-deoxyglucose with 1 mg/kg glucose (glucose groups) or without glucose (basal groups), and the glucose uptake rates for the (**A**) TA, (**B**) EDL, (**C**) SOL, (**D**) CM, (**E**) BAT, (**F**) WAT, and (**G**) WB were determined (*P < 0.05 and **P < 0.01 for glucose-KO or basal-KO vs. glucose-WT or basal-WT, respectively; *n* = 5 to 6 mice for each group). (**H** and **I**) Blood was collected from each mouse at baseline and at various time points to compare the serum glucose level (mean ± SEM) between the WT and KO groups. The overall comparison of the datasets was based on two-way ANOVA (*P*-value in panel), which was followed by the Bonferroni test to compare data for the WT and KO groups at each time point (*P < 0.05).

We examined the effects of r-m-ApoA-IV on glucose uptake in the adipose and muscle tissues of WT and ApoA-IV-KO mice. After fasting overnight, anesthetized mice were pretreated with r-m-ApoA-IV or saline for 2 h before being treated with a mixture of [^3^H]-2-deoxyglucose with or without glucose and/or insulin. At 45 min post-treatment, the level of tritiated tracer in various tissues was determined, and the GUR was calculated as the amount of glucose in nanograms per milligram body weight per minute (ng/mg/min) for the various tissue samples collected (Fig. 3A–H). The whole body (WB) and total muscle GURs were calculated as the sum of the GURs of all the tissue types collected and all of the muscle samples collected, respectively. In the WT mice that received both the r-m-ApoA-IV pretreatment and the glucose treatment, the GURs for the WB, intrascapular brown adipose tissue (BAT), visceral white adipose tissue (WAT), and cardiac muscle (CM) were significantly higher than that of the mice that received the saline pretreatment and glucose alone (Fig. 3E‒H), and the GURs for the CM and WB were also significantly higher than those of the mice that received the saline pretreatment followed by treatment with glucose and insulin (Fig. 3G and H), whereas the GURs for the red skeletal muscle (RSM) from the soleus (SOL), the white skeletal muscle (WSM) from EDL and the mixed from the tibialis anterior (TA, mixed WSM and RSM), and the total muscle were significantly higher than those of the mice that received the saline pretreatment and glucose alone, but were not significantly greater than those of the mice that received the saline pretreatment followed by treatment with glucose and insulin (Fig. 3A,C, and D). No significant difference in GUR was observed for WSM from the extensor digitorum longus (EDL) in WT mice that received the r-m-ApoA-IV pretreatment and glucose, compared to that of mice that received the saline pretreatment and glucose. Figure 4 shows that r-m-ApoA-IV pretreatment produced a small significant increase in level of endogenous insulin in mice treated with or without glucose, compared to the saline pretreated mice (*P* < 0.05), that was significantly smaller than that observed in the mice treated with insulin without r-m-ApoA-IV pretreatment at 5‒15 min post-treatment (*P* < 0.05). Therefore, although the contribution of insulin-mediated effects cannot be discounted, these data suggest that the observed increases in the GURs for WAT, BAT, CM, and WB in WT mice that received both r-m-ApoA-IV and glucose were mediated by an insulin-independent mechanism.

### Efficient glucose uptake in skeletal muscle requires ApoA-IV

We compared the GURs of WT and ApoA-IV-KO mice that received glucose or saline. No significant difference was observed between the GURs of tissues from WT and ApoA-IV-KO mice without glucose, with the exception of the TA (Fig. 4A–G). The GURs for TA, EDL, and SOL in the ApoA-IV-KO mice that received glucose were significantly lower (*P* < 0.05) than those of the WT mice that received glucose, whereas the GURs for WAT, BAT, CM, and WB were not (*P* ≥ 0.05). As shown in Fig. 4, the blood glucose levels in the WT and ApoA-IV-KO mice were not significantly different for either the basal or glucose groups (*P* ≥ 0.05), with the exception of the 5 min time point for the glucose groups (Fig. 4H and I). Although the results of the glucose uptake analysis in WT mice (Fig. 3) showed that the enhancement of glucose uptake by ApoA-IV was greater for WAT, BAT, and CM than for WSM, RSM, or mixed skeletal muscle, the analysis in ApoA-IV-KO mice (Fig. 4) suggested that the extent to which ApoA-IV is required for efficient glucose uptake is greater for WSM, RSM, and mixed skeletal muscle than for WAT, BAT, and CM.

### ApoA-IV upregulates GLUT4 translocation in 3T3-L1 adipocytes

**Figure 5:**
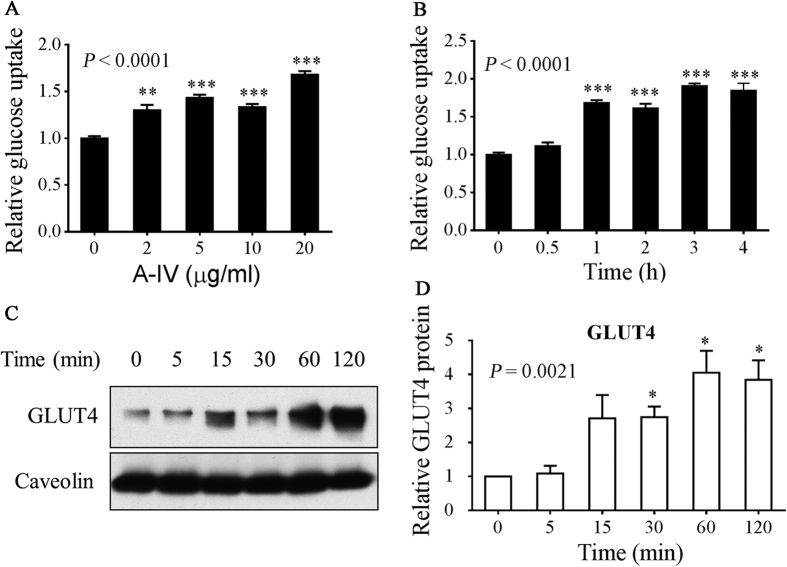
Effect of ApoA-IV on glucose uptake and GLUT4 translocation in adipocytes. Glucose uptake in 3T3-L1 adipocytes was assessed following treatment with (**A**) 0‒20 μg/mL m-r-ApoA-IV for 1 h or (**B**) 20 μg/mL m-r-ApoA-IV for 0‒4 h. To evaluate GLUT4 translocation, 3T3-L1 adipocytes were treated with 20 μg/mL m-r-ApoA-IV, and the membrane protein fraction was isolated from whole cell lysates at 0‒120 min. (**C**) Membrane proteins were subjected to SDS-PAGE and western blotting using anti-GLUT4 and anti-caveolin antibodies, and (**D**) the results were quantified by densitometry. Data are expressed as the mean fold increase ± SEM of three independent experiments. Differences in relative glucose uptake and relative GLUT4 protein levels, compared to vehicle control, were evaluated using one-way ANOVA (*P*-value in panel), which was followed by the Dunnett test to compare glucose uptake at different ApoA-IV dosages or the GLUT4 protein level at different time points relative to baseline (*P < 0.05, **P < 0.01, ***P < 0.001).

We also assessed glucose uptake in 3T3-L1 adipocytes treated with ApoA-IV in the absence of insulin. As shown in Fig. 5(A and B), relative glucose uptake increased over time, and it increased with increasing r-m-ApoA-IV, compared with the vehicle control. We also examined the translocation of GLUT4-containing vesicles in 3T3-L1 adipocytes, which is a process that is actively involved in the internalization of glucose from serum^15^. Cell membrane protein fractions were isolated from whole cell lysates following treatment with r-m-ApoA-IV, and the level of GLUT4 was assessed by western blotting. Treatment with 2‒20 μg/mL r-m-ApoA-IV for 30 min significantly increased glucose uptake, compared to the vehicle control, and treatment 20 μg/mL r-m-ApoA-IV increased glucose uptake from 1‒4 h post-treatment (Fig. 5A and B, respectively; *P* < 0.0001 for both). Western blotting analysis showed that, following treatment with 20 μg/mL r-m-ApoA-IV, the level of GLUT4 in the membrane protein fraction significantly increased at 15‒120 min post-treatment, relative to the level of caveolin (Fig. 5C and D; *P* < 0.0001). These data suggest that ApoA-IV increases glucose uptake in adipocytes by stimulating GLUT4 translocation via an insulin-independent mechanism.

### ApoA-IV activates Akt signaling in 3T3-L1 adipocytes

**Figure 6:**
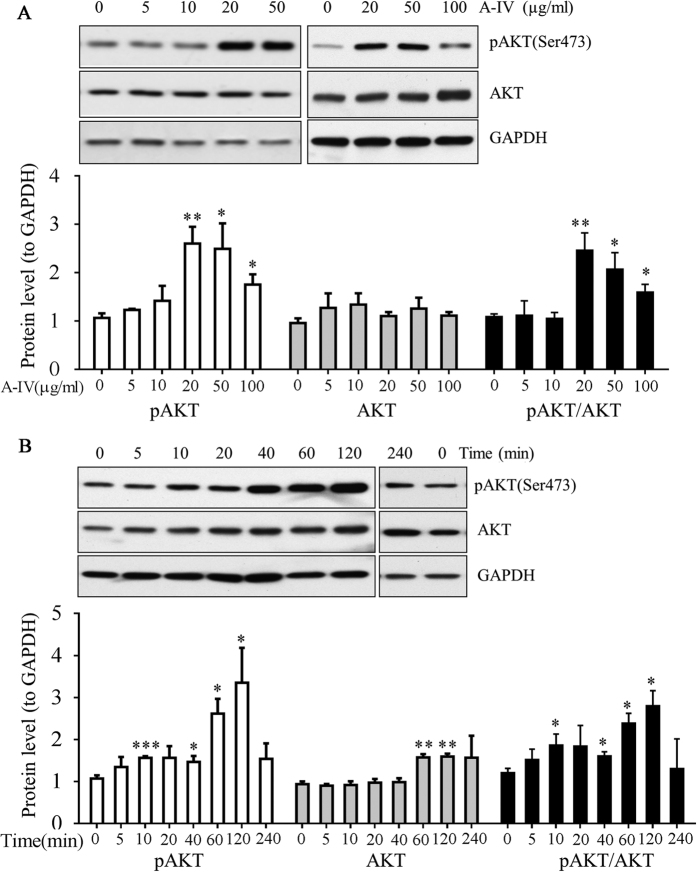
Dose- and time-dependent effects of ApoA-IV on Akt phosphorylation. The 3T3-L1 adipocytes were pretreated with (**A**) the indicated dose of m-r-ApoA-IV for 30 min, or (**B**) 20 μg/mL m-r-ApoA-IV for the indicated time in the absence of insulin. Cell lysates were subjected to SDS-PAGE and western blotting using anti-pSer473-Akt, anti-Akt, and anti-GAPDH (loading control) antibodies. A representative blot from at least four independent experiments is shown. Immunoblotting results were quantified by densitometry, and the data are expressed as the mean fold increase ± SEM. Differences in relative protein levels in ApoA-IV treated cells, compared to vehicle control (*P < 0.05, **P < 0.01, ***P < 0.001).

**Figure 7:**
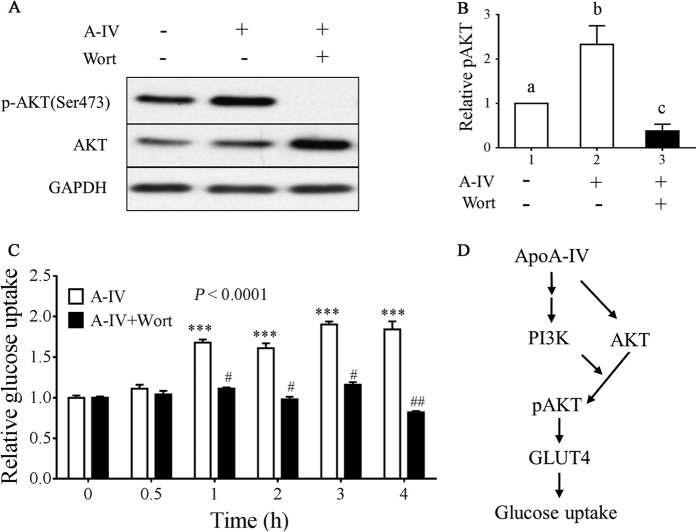
Effects of PI3K inhibitor wortmannin on ApoA-IV stimulated Akt activation and glucose uptake. (**A** and **B**) 3T3-L1 adipocytes were pretreated with or without 100 nM wortmannin (wort) for 30 min before treatment with 20 μg/mL r-m-ApoA-IV for 40 min. Whole cell lysates were subjected to SDS-PAGE and western blotting using anti-pSer473-Akt, anti-Akt, and anti-GAPDH (loading control) antibodies, as shown in (**A**) a representative blot from one of at least three independent experiments. Immunoblotting results were quantified by densitometry, and the data are expressed as fold increase ± SEM (*P < 0.05 vs. vehicle control). (**C**) The PI3K inhibitor wortmannin inhibited ApoA-IV stimulated glucose uptake. 3T3-L1 adipocytes were pretreated with 100 nM wortmannin (A-IV + wort group) or without wortmannin (A-IV group) for 30 min before treatment with 20 μg/mL ApoA-IV, and glucose uptake was assessed over time. Data are presented as the mean fold increase ± SEM of three independent experiments. Differences in relative glucose uptake for the A-IV and A-IV + wort groups, compared to vehicle control, were evaluated using one-way ANOVA, which was followed by the Dunnett test to compare data at different time points relative to baseline (***P < 0.001). The A-IV and A-IV + wort datasets were compared using two-way ANOVA, which was followed by Bonferroni test to evaluate differences between the data for the A-IV and A-IV + wort groups at each time point (^#^*P* < 0.01 and ^##^*P* < 0.001). (**D**) Diagram depicting the proposed roles of ApoA-IV in the regulation of glucose uptake in mouse adipocytes. ApoA-IV induces both the expression and activation of Akt via PI3K and insulin mediated signaling, which upregulates GLUT4 translocation, thereby enhancing glucose internalization.

To examine the effect of ApoA-IV on Akt signaling in adipocytes, we performed a time course analysis of the level of phospho-Akt following treatment with r-m-ApoA-IV using western blotting. Treatment with 20, 50, and 100 μg/mL r-m-ApoA-IV for 30 min significantly increased the level of phospho-Akt, compared to the baseline phospho-Akt level (Fig. 6A; *P* < 0.05), and treatment with 20 μg/mL r-m-ApoA-IV significantly increased the level of phospho-Akt at 10, 20, 40, 60, and 120 min post-treatment, compared to the level of phospho-Akt at baseline (Fig. 6B; *P* < 0.05). We also found that treatment with 20 μg/mL r-m-ApoA-IV significantly increased the level of Akt protein at 60 and 120 min post-treatment (Fig. 6B; *P* < 0.05), which suggested that Apo-A-IV upregulated both the activation and expression of Akt. In the Akt mediated uptake of glucose in adipocytes in response to insulin, PI3K functions as an activator of Akt signaling^5^. Therefore, we examined glucose uptake and Akt phosphorylation in 3T3-L1 adipocytes following r-m-ApoA-IV treatment in the presence and absence of the PI3K inhibitor, wortmannin. We found that, in 3T3-L1 adipocytes treated with 20 μg/mL r-m-ApoA-IV for 40 min, phosphorylation of Akt was abolished in the presence of 100 nM wortmannin (Fig. 7A and B), and we observed that relative glucose uptake in ApoA-IV treated cells was significantly reduced by treatment with 100 nM wortmannin at 1, 2, 3, and 4 h post-treatment, compared to that in cells treated with ApoA-IV alone (Fig. 7C; *P* < 0.001). These results indicated that ApoA-IV stimulates the phosphorylation of Akt and glucose uptake was mediated via PI3K signaling. These data combined with those of our other experiments suggest that ApoA-IV stimulates the phosphorylation and expression of Akt, at least in part via PI3K, which increases the uptake of glucose in adipocytes through the upregulation of GLUT4 translocation, as shown in Fig. 7D.

## Discussion

The primary goal of treatment for IR is to reduce patients’ fasting plasma glucose levels and improve insulin resistance in an effort to inhibit disease progression^13^. In our previous studies, we showed that, in *KKAy* diabetic mice, pretreatment with exogenous ApoA-IV before administering glucose lowered plasma glucose by reducing hepatic glucose production and enhancing insulin secretion^8^,^9^. In our current study, we investigated the effect of ApoA-IV on insulin sensitivity in WT (C57BL/6J strain) and *KKAy* diabetic mice. We found that treatment with ApoA-IV lowered fasting blood glucose in both WT and *KKAy* diabetic mice by increasing glucose uptake in CM, WAT, and BAT through a mechanism that is at least partially independent of insulin.

We examined the pathways underlying the effects of ApoA-IV in 3T3-L1 mouse adipocytes in cell culture to identify the biochemical mechanisms involved. Cell culture experiments showed that ApoA-IV improved glucose uptake in 3T3-L1 adipocytes in the absence of insulin by upregulating GLUT4 translocation through the PI3K mediated activation of Akt signaling pathways. The difference between the magnitude of the effect of ApoA-IV on adipose tissues in mice and its effects on 3T3-L1 cells is likely the result of changes in serum insulin that influenced glucose uptake *in vivo*. The results of our current study and our previous finding that ApoA-IV treatment enhanced pancreatic insulin secretion in mice suggests that ApoA-IV acts directly upon adipose tissue to improve glucose uptake and indirectly via elevated serum insulin in response to increased blood glucose.

Akt contributes to the regulation of GLUT4-mediated glucose uptake in muscle cells and adipocytes in response to insulin. The initial events in the internalization of glucose containing vesicles include the binding of insulin to the insulin receptor and the activation of insulin receptor tyrosine kinase. The subsequent phosphorylation of insulin receptor substrates (IRSs) and their interaction with several downstream targets that stimulate the translocation of GLUT4^4^. Upon phosphorylation, IRS-1 is bound by PI3K, which plays a primary role in Akt signaling pathways^16^. The PI3K-Akt signaling axis is also activated by other factors, including B and T cell receptors, cytokine receptors, receptor tyrosine kinases, integrins, and G-protein-coupled receptors^17^. However, our current finding is the first report of ApoA-IV as a regulator of glucose homeostasis in adipocytes via PI3K-Akt signaling.

Considerable evidence suggests that low-grade inflammation leads to the hyperphosphorylation of IRSs and multiple serine kinases, which contributes to reduced Akt activation in the muscle cells of patients with IR^18^,^19^. Our results showed that the activation of Akt and the upregulation of GLUT4 translocation and glucose uptake each occurred in two phases, with Akt phosphorylation peaking at 10‒20 min and 1‒2 h, peak GLUT4 translocation occurring at 15 and 60 min, and glucose uptake peaking at 1 and 3 h following ApoA-IV treatment. In our previous studies, we found that ApoA-IV was internalized by human liver (HepG2) and kidney (HEK-293) cells, and colocalized with the NR1D1 or NR4A1 nuclear receptor, which suppressed the transcriptional activity of gluconeogenic genes^8^,^20^. We speculate that the early intercellular response to ApoA-IV is mediated by a receptor that has yet to be identified that activates the PI3K-Akt cascade, whereas the late response is likely mediated by downstream targets of the PI3K-Akt or ApoA-IV-NR1D1/NR4A1 pathways. Given the development of hyperglycaemia, hyperinsulinaemia, and impaired glucose uptake in skeletal muscle observed in *Akt* knockout mice^21^, it is possible that the activation of Akt signaling also affects downstream targets involved in gene expression. Such downstream effects might explain the increase in *Akt* expression that we observed in 3T3-L1 adipocytes following ApoA-IV treatment, as well as our previously reported observation of an increase in insulin secretion by ApoA-IV treated pancreatic islets in response to glucose *in vitro*.

In conclusion, we investigated the effects of treatment with exogenous ApoA-IV on glucose homeostasis in WT and diabetic mice and in cultured adipocytes. We found that the ApoA-IV treatment contributed to the regulation of blood sugar by acting directly upon adipose tissue to improve glucose uptake. With regard to the relevance of our findings to energy homeostasis as a whole, it is important to consider that the production of ApoA-IV by enterocytes is stimulated only by active fat absorption, whereas neither the production nor the secretion of ApoA-IV are stimulated by carbohydrate or protein absorption, which indicates that ApoA-IV serves as a bridge between lipid metabolism and carbohydrate metabolism. Our findings warrant future studies to identify the cellular receptor for ApoA-IV in adipocytes, possible links between the ApoA-IV-NR1D1/NR4A1 pathway and PI3K-Akt signaling pathways, and the downstream targets of PI3K-Akt mediated signal transduction that underlie the upregulation of GLUT4 translocation and glucose uptake in adipocytes to assess their potential value as therapeutic targets for the treatment of IR and T2DM.

## Additional Information

**How to cite this article:** Li, X. *et al*. ApoA-IV improves insulin sensitivity and glucose uptake in mouse adipocytes via PI3K-Akt Signaling. *Sci. Rep.* **7**, 41289; doi: 10.1038/srep41289 (2017).

**Publisher's note:** Springer Nature remains neutral with regard to jurisdictional claims in published maps and institutional affiliations.
